# Supplementary material for: Temporal morphogen gradient-driven neural induction shapes single expanded neuroepithelium brain organoids with enhanced cortical identity
Source: Nat Commun. 2023 Nov 28;14:7361. doi: 10.1038/s41467-023-43141-1 (PMC10684874; doi:10.1038/s41467-023-43141-1)
Supplement: Supplementary file 1 — Supplementary Information [file 41467_2023_43141_MOESM1_ESM.pdf]

Temporal morphogen gradient-driven neural induction shapes single expanded neuroepithelium brain organoids with enhanced cortical identity

Anna Pagliaro[1], Roxy Finger[1], Iris Zoutendijk[1], Saskia Bunschuh[1], Hans Clevers[1,2,3,4], Delilah Hendriks[1,2,3*#], Benedetta Artegiani[1*#]

[1]The Princess Maxima Center for Pediatric Oncology, Utrecht, The Netherlands
[2]Hubrecht Institute, Royal Netherlands Academy of Arts and Sciences, Utrecht, The Netherlands
[3]Oncode Institute, Utrecht, The Netherlands
[4]Pharma, Research and Early Development (pRED) of F. Hoffmann-La Roche Ltd, Basel, Switzerland

[*]These authors jointly supervised this work

[#]Corresponding authors:
d.hendriks@hubrecht.eu, b.a.artegiani@prinsesmaximacentrum.nl

This file contains:
Supplementary Figures 1-13
Supplementary Tables 1-3

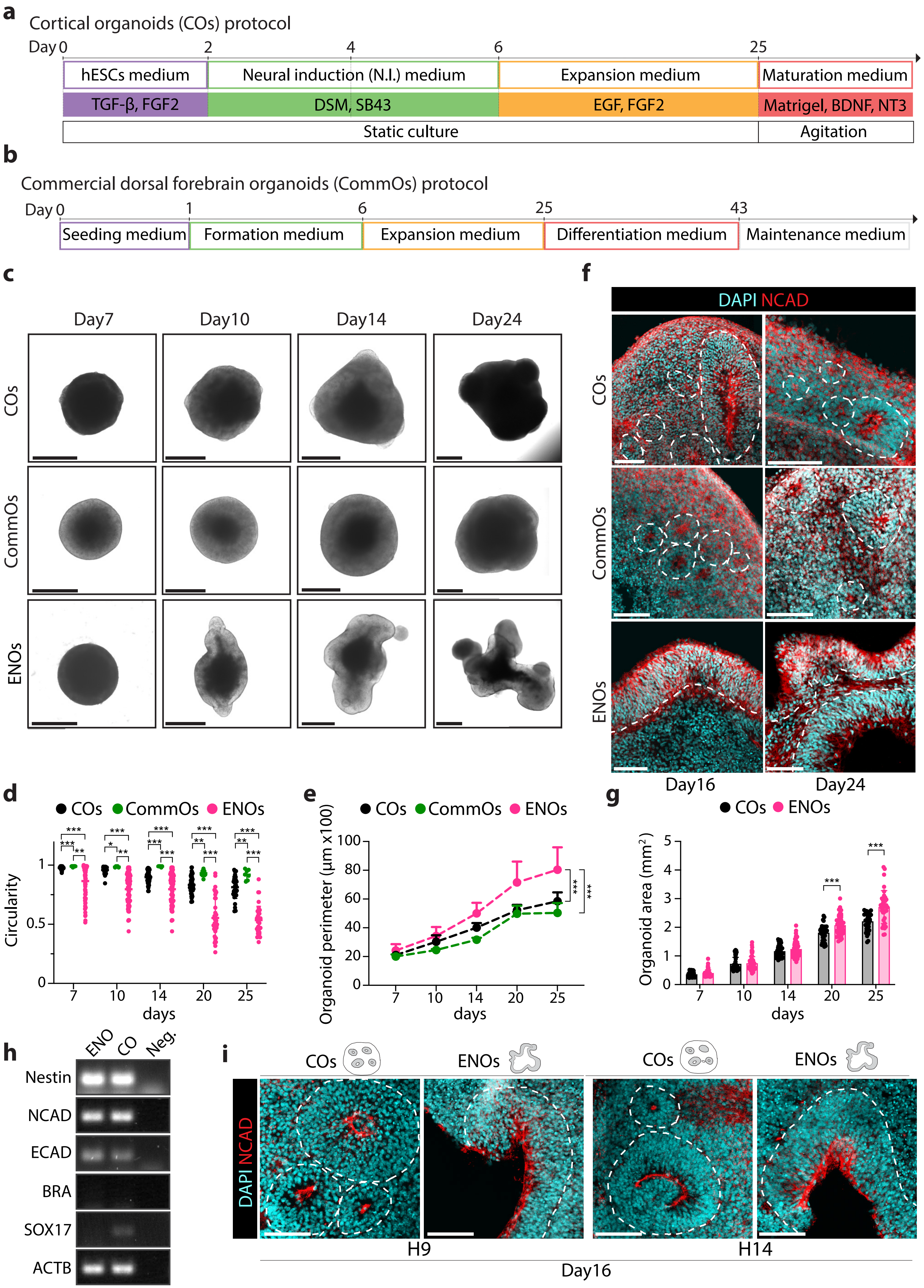

a
Cortical organoids (COs) protocol
Day 0 2 4 6 25
hESCs medium
Neural induction (N.I.) medium
Expansion medium
Maturation medium
TGF-β, FGF2
DSM, SB43
EGF, FGF2
Matrigel, BDNF, NT3
Static culture
Agitation
b
Commercial dorsal forebrain organoids (CommOs) protocol
Day 0 1 6 25 43
Seeding medium
Formation medium
Expansion medium
Differentiation medium
Maintenance medium
c
Day7 Day10 Day14 Day24
COs
CommOs
ENOs
f
DAPI NCAD
COs
CommOs
ENOs
Day16 Day24
d
COs CommOs ENOs
Circularity
days
e
COs CommOs ENOs
Organoid perimeter (µm x100)
days
g
COs ENOs
Organoid area (mm²)
days
h
ENO CO Neg.
Nestin
NCAD
ECAD
BRA
SOX17
ACTB
i
COs ENOs COs ENOs
DAPI NCAD
H9
H14
Day16

Supplementary Figure 1. Comparative analysis of ENOs with cortical organoid protocols

a. Schematic illustration of timeline and protocol for generating cortical organoids (COs). **b.** Schematic illustration of timeline and protocol for generating commercial dorsal forebrain organoids (CommOs). **c.** Representative brightfield images of COs, CommOs, and ENOs at the indicated timepoints. Scale bars = 500 µm. **d.** Quantification of the circularity of COs, CommOs, and ENOs measured at the indicated time points. Each dot represents an organoid and mean ± SD is plotted. **$p < 0.01$; ***$p < 0.001$; Two-tailed unpaired t-test. **e.** Quantification of the organoid perimeter of COs, CommOs, and ENOs measured at the indicated time points. Mean ± SD is plotted. ***$p < 0.001$; Two-tailed unpaired t-test. **f.** Representative immunofluorescence images of COs, CommOs and ENOs at day 16 and 24 stained for NCAD with DAPI counterstained. Dashed lines delineate rosettes and neuroepithelium structures and in the different organoids. Scale bars = 100 µm. **g.** Quantification of the area of COs and ENOs measured at the indicated time points. Mean ± SD is plotted (n z 7 organoids per timepoint, with n z 3 organoids analysed per batch). ***$p < 0.001$; Two-tailed unpaired t-test. **h.** qPCR analysis of selected neuroectoderm (N-CAD and NESTIN), non-neural epithelium (ECAD), mesoderm (BRACH), and endoderm (SOX17) markers in day 16 ENOs and COs. Neg. = negative H2O control. **i.** Representative immunofluorescence images of COs and ENOs at day 16 formed with H9 and H14 hESC lines stained for NCAD with DAPI counterstained. Dashed lines delineate basal perimeter of rosettes and neuroepithelium structures in the different organoids. Scale bars = 100 µm. For **d-e, g**, a detailed description of how many organoids and batches were analyzed is described in **Supplementary Table 3**. Images in **c, f** and **i** are representative of 3 independent experiments. Exact sample size and exact P values are provided in Source Data. Source data are provided as Source data file.

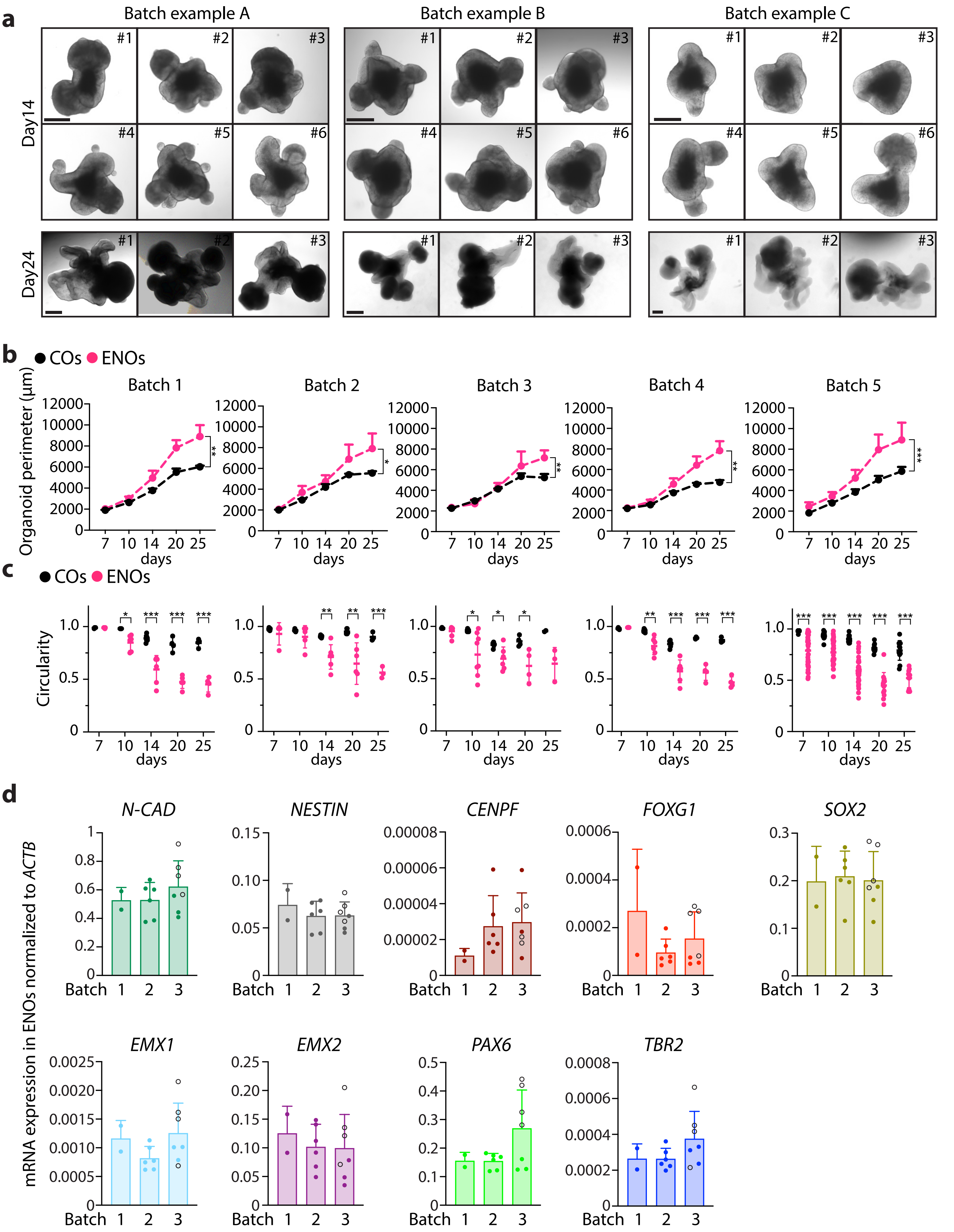

a
Batch example A
Batch example B
Batch example C
Day14
Day24
#1
#2
#3
#4
#5
#6
b
COs ENOs
Batch 1
Batch 2
Batch 3
Batch 4
Batch 5
Organoid perimeter (µm)
days
c
COs ENOs
Circularity
days
d
N-CAD
NESTIN
CENPF
FOXG1
SOX2
EMX1
EMX2
PAX6
TBR2
mRNA expression in ENOs normalized to ACTB
Batch 1 2 3

Supplementary Figure 2. Batch-to-batch reliability of ENO formation

a. Representative brightfield images of day 14 and 24 ENOs for n = 3 batches from the H1 hESC line. Scale bars = 500 μm. **b.** Quantification of the organoid perimeter of COs and ENOs measured at the indicated timepoints for n = 5 batches. Mean ± SD is plotted. **p* < 0.05; ***p* < 0.01; ****p* < 0.001; Two-tailed unpaired t-test. **c.** Quantification of the organoid circularity of COs and ENOs measured at the indicated timepoints for the same n = 5 batches mentioned in **b**. Mean ± SD is plotted. **p* < 0.05; ***p* < 0.01; ****p* < 0.001; Two-tailed unpaired t-test. **d.** qPCR analysis displaying expression variation of selected neuroectoderm (*N-CAD* and *NESTIN*), forebrain progenitors (*FOXG1, SOX2, EMX1, EMX2, PAX6*), cell cycle-related gene *CENPF*, and intermediate progenitor marker *TBR2* (normalized over *ACTB*) in Day 16 ENOs and no SB43 organoids (empty dots) for n = 3 batches. Exact sample size for **b** and **c** and exact P values are provided in Source Data. Source data are provided as Source data file.

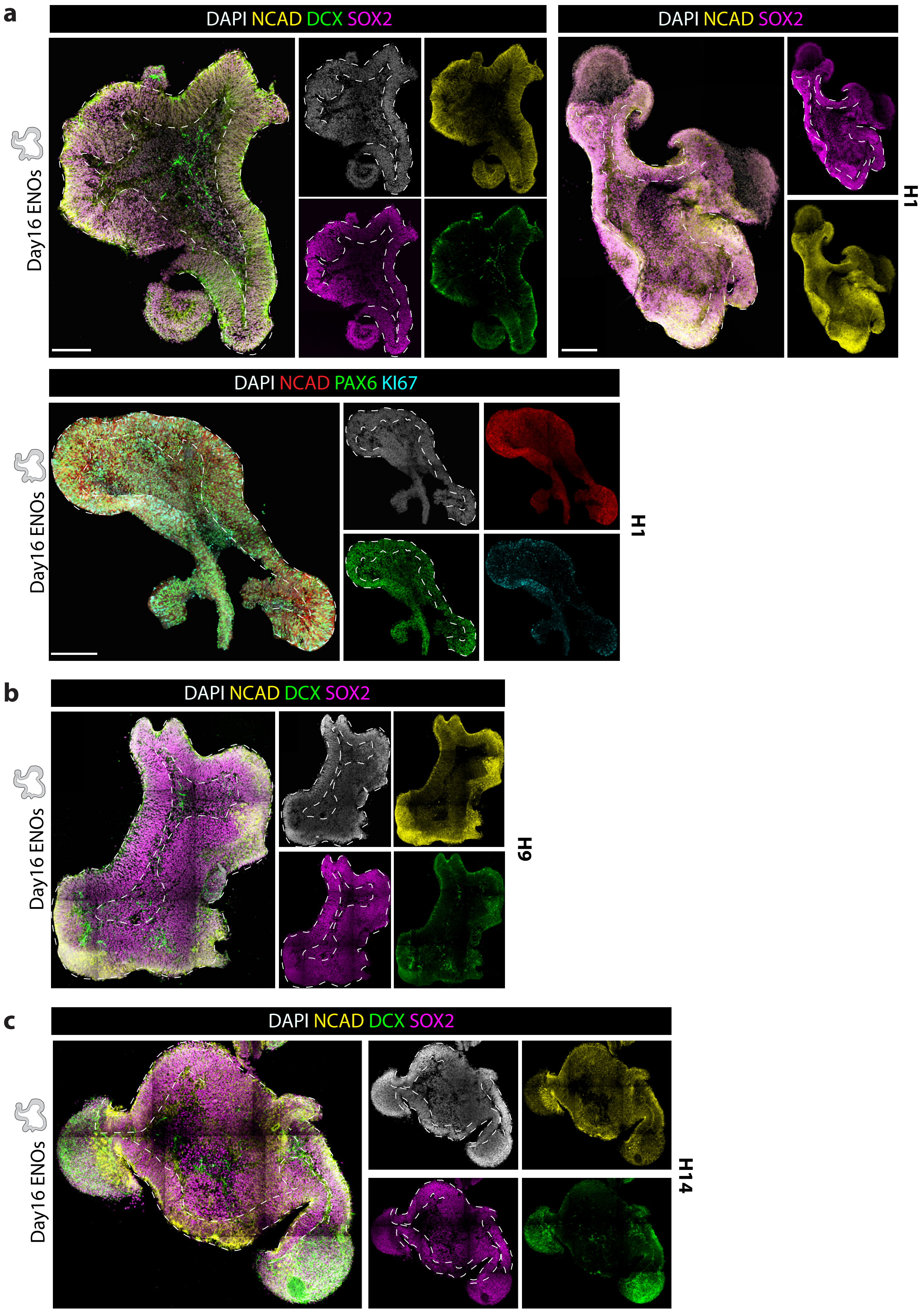

a
DAPI NCAD DCX SOX2
DAPI NCAD SOX2
Day16 ENOs
H1
DAPI NCAD PAX6 KI67
Day16 ENOs
H1
b
DAPI NCAD DCX SOX2
Day16 ENOs
H9
c
DAPI NCAD DCX SOX2
Day16 ENOs
H14

Supplementary Figure 3. Characterization of the tissue architecture of whole ENOs

a. Representative immunofluorescence images of whole H1 ENOs at day 16 stained for NCAD, DCX and SOX2 (left), and NCAD and SOX2 (right), and for NCAD, PAX6 and KI67 (bottom) with DAPI counterstained. **b.** Representative immunofluorescence image of a whole H9 ENO at day 16 stained for NCAD, DCX, and SOX2 with DAPI counterstained. **c.** Representative immunofluorescence image of a whole H14 ENO at day 16 stained for NCAD, DCX, and SOX2 with DAPI counterstained. Dashed delineate the apical and basal perimeters of the neuroepithelium structures. Scale bars are 200 μm. Images are representative of n=3 independent experiments.

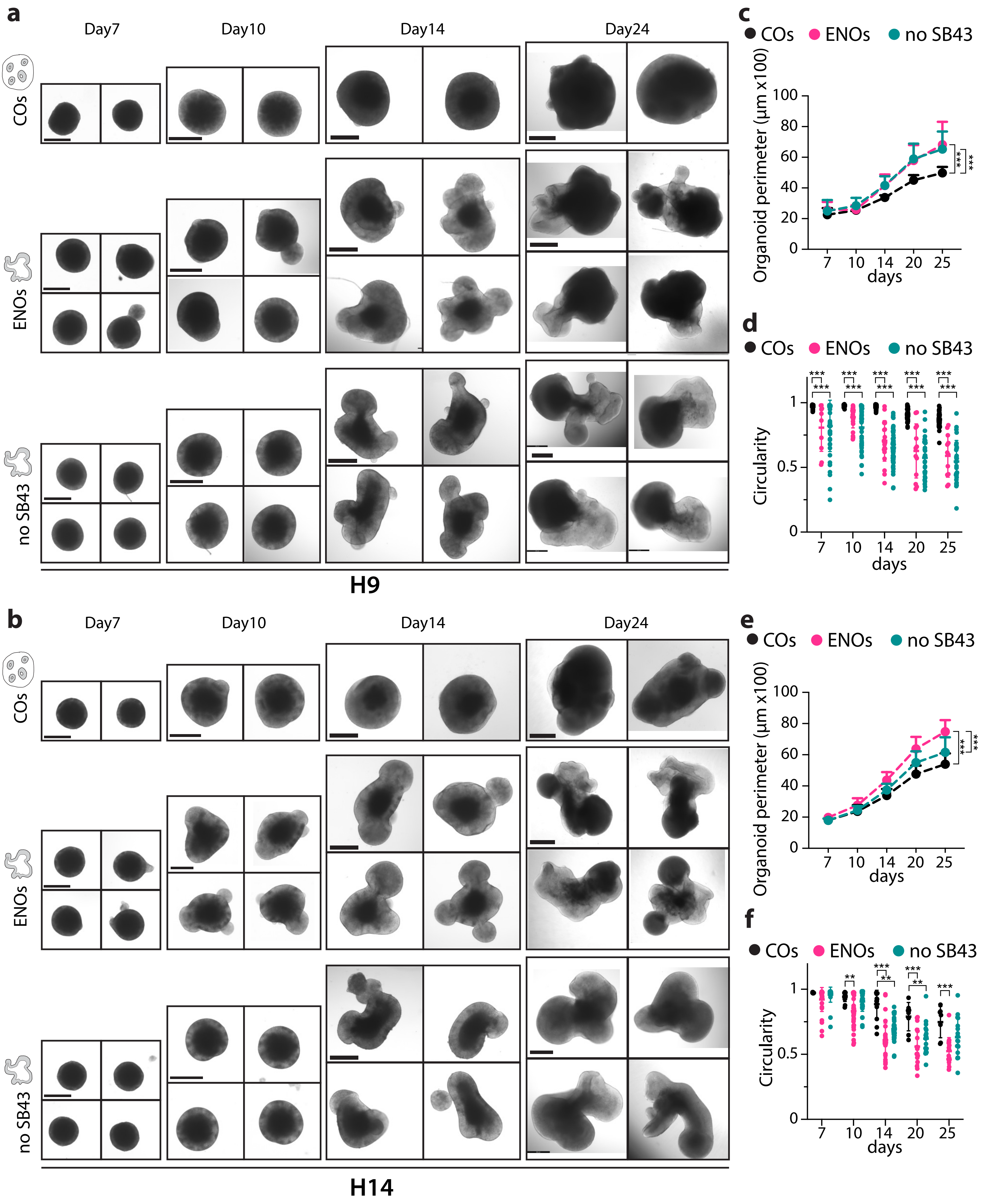
a
Day7
Day10
Day14
Day24
COs
ENOs
no SB43
H9
b
Day7
Day10
Day14
Day24
COs
ENOs
no SB43
H14
c
COs ENOs no SB43
Organoid perimeter (µm x100)
100
80
60
40
20
0
7 10 14 20 25
days
***
***
d
COs ENOs no SB43
Circularity
1
0.5
0
7 10 14 20 25
days
e
COs ENOs no SB43
Organoid perimeter (µm x100)
days
f
COs ENOs no SB43
Circularity
days

Supplementary Figure 4. Reproducibility of ENO formation across different hESC lines under different TGF-β gradients

a. Representative brightfield images of COs, ENOs, and no SB43 organoids at the indicated timepoints formed using the H9 hESC line. Scale bar = 500 μm. **b.** Quantification of the organoid perimeter of COs, ENOs, and no SB43 measured at the indicated time points, formed using the H9 hESC line. Mean ± SD is plotted. ***$p < 0.001$; Two-tailed unpaired t-test. **c.** Quantification of the organoid circularity of COs, ENOs, and no SB43 organoids measured at the indicated time points, formed using the H9 hESC line. Each dot represents an organoid and mean ± SD is plotted. ***$p < 0.001$; Two-tailed unpaired t-test. **d.** Representative brightfield images of COs, ENOs and no SB43 organoids at the indicated timepoints formed using the H14 hESC line. Scale bar = 500 μm. **e.** Quantification of the organoid perimeter of COs, ENOs and no SB43 organoids measured at the indicated time points, formed using the H14 hESC line. Mean ± SD is plotted. ***$p < 0.001$; Two-tailed unpaired t-test. **f.** Quantification of the organoid circularity of COs, ENOs, and no SB43 measured at the indicated time points, formed using the H14 hESC line. Each dot represents an organoid and mean ± SD is plotted. **$p < 0.01$; ***$p < 0.001$; Two-tailed unpaired t-test. For **b-c, e-f**, a detailed description of how many organoids and batches were analyzed is described in **Supplementary Table 3**. Images in **a** and **d** are representative of n=3 independent experiments. Exact sample size for **b, c, d** and **f** and exact P values are provided in Source Data. Source data are provided as Source data file.

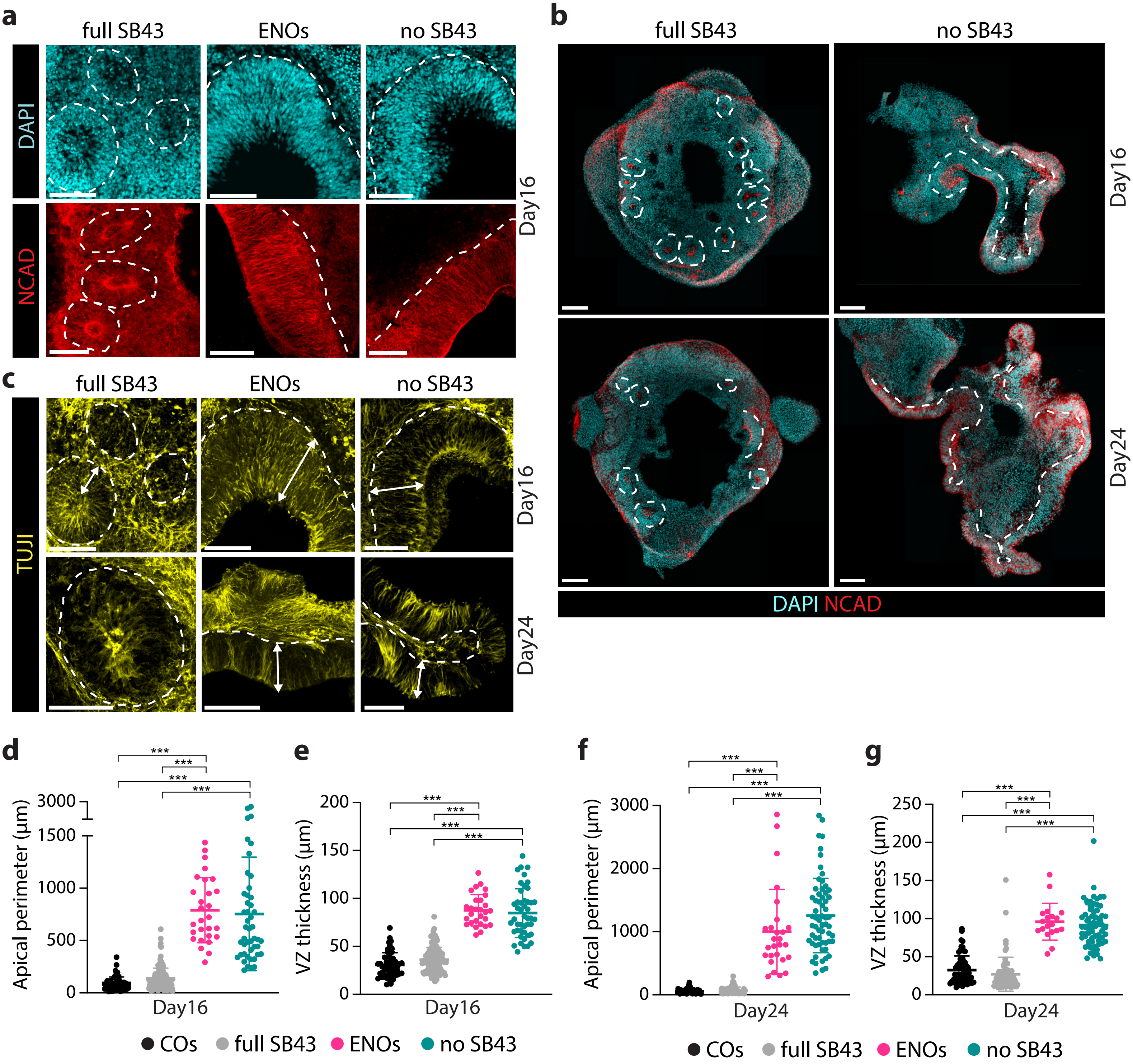

a
full SB43
ENOs
no SB43
DAPI
NCAD
Day16
b
full SB43
no SB43
Day16
Day24
DAPI NCAD
c
full SB43
ENOs
no SB43
TUJI
Day16
Day24
d
Apical perimeter (µm)
Day16
e
VZ thickness (µm)
Day16
COs full SB43 ENOs no SB43
f
Apical perimeter (µm)
Day24
g
VZ thickness (µm)
Day24
COs full SB43 ENOs no SB43

Supplementary Figure 5. Cellular characterization of organoids generated with different TGF-β gradients

a. Representative immunofluorescence images of day 16 full SB43, ENOs, and no SB43 organoids stained for NCAD with DAPI counterstained. Scale bars = 100 μm. **b.** Representative immunofluorescence image of whole day 16 and 24 full SB43 and no SB43 whole organoids stained for NCAD with DAPI counterstained. Scale bars = 200 μm. **c.** Representative immunofluorescence images of day 16 and 24 full SB43, ENOs, and no SB43 stained for TUJ1. Arrows point at the edges of the ventricular zone. Scale bars = 100 μm. **d.** Quantification of the apical perimeter of rosettes/neuroepithelium structures of day 16 COs, full SB43, ENOs, and no SB43 based on NCAD+ epithelium. Each dot represents individual rosettes/neuroepithelium structures and mean ± SD is plotted. COs: n = 9, full SB43: n = 13, ENOs: n = 12, no SB43: n = 12 organoids; n = 3, 4, 4, 4 batches. ***$p < 0.001$; Two-tailed unpaired t-test. **e.** Quantification of VZ thickness of rosettes/neuroepithelium structures of day 16 COs, full SB43, ENOs, and no SB4. Each dot represents the average of three measurements for each individual neuroepithelium/rosette structure and mean ± SD is plotted. Sample size as in d. ***$p < 0.001$; Two-tailed unpaired t-test. **f.** Quantification of the apical perimeter of rosettes/neuroepithelium structures of day 24 COs, full SB43, ENOs, and no SB43. Each dot represents individual rosettes/neuroepithelium structures and mean ± SD is plotted. COs: n = 10, full SB43: n = 14, ENOs: n = 12, no SB43: n = 18 organoids; n = 3, 4, 4, 4 batches. ***$p < 0.001$; Two-tailed unpaired t-test. **g.** Quantification of VZ thickness of rosettes/neuroepithelium structures of day 24 COs, full SB43, ENOs and no SB43. Each dot represents average of three measurements for each individual neuroepithelium/rosette structure and mean ± SD is plotted. Sample size as in f. ***$p < 0.001$; Two-tailed unpaired t-test. Images in **a, b** and **c** are representative of n=3 independent experiments. Dashed lines in **a, b** and **c** delineate basal perimeter of rosettes/neuroepithelium structures in the different organoids. Exact sample size for **d, e, f** and **g** and exact P values are provided in Source Data. Source data are provided as Source data file.

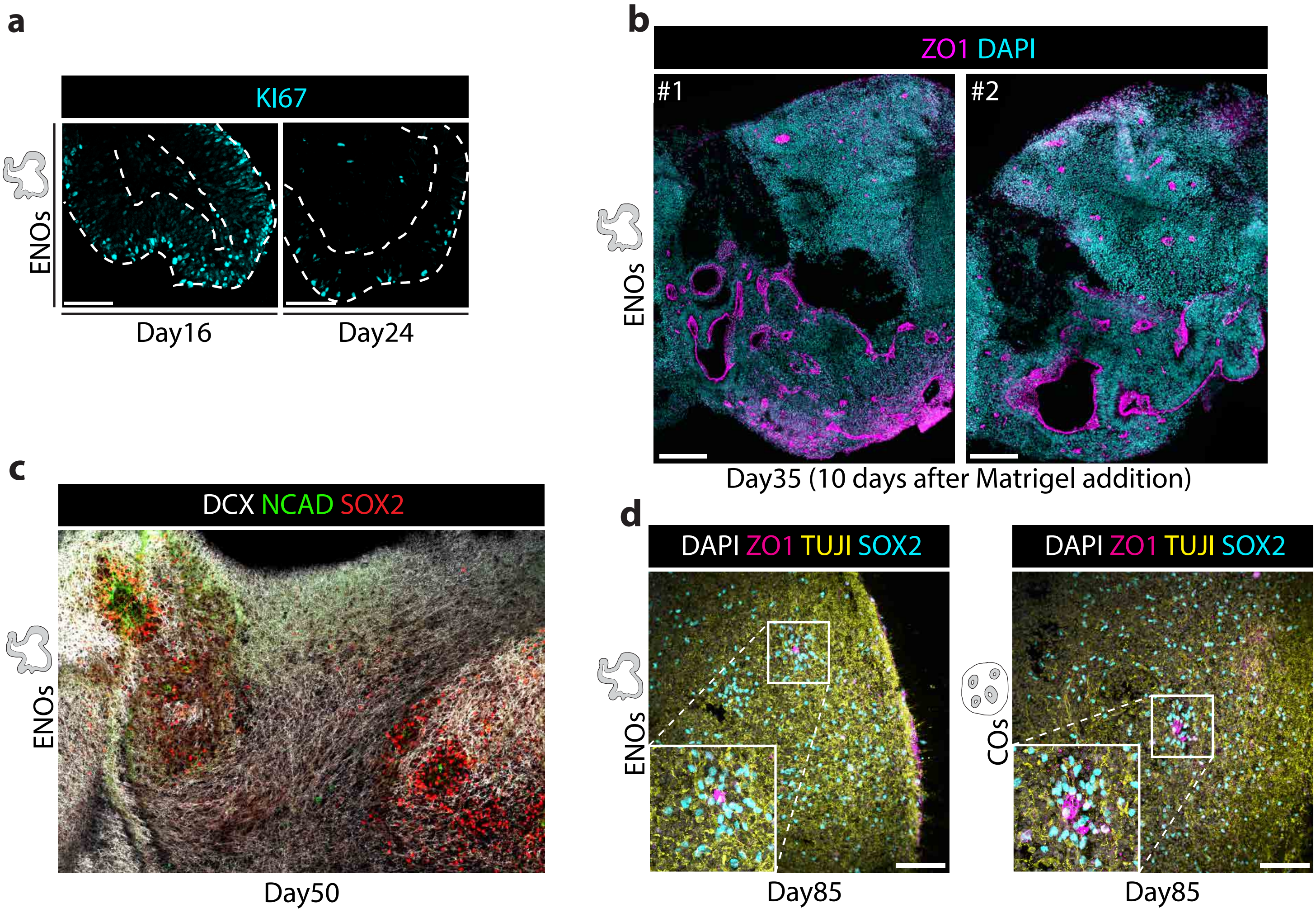
a
KI67
ENOs
Day16
Day24
b
ZO1 DAPI
#1
#2
ENOs
Day35 (10 days after Matrigel addition)
c
DCX NCAD SOX2
ENOs
Day50
d
DAPI ZO1 TUJI SOX2
ENOs
Day85
DAPI ZO1 TUJI SOX2
COs
Day85

Supplementary Figure 6. Proliferation in ENOs and architectural structures at later timepoints

a. Representative immunofluorescence images of day 16 and 24 ENOs stained for KI67. Dashed lines delineate apical and basal perimeter of the neuroepithelium structures. Scale bars = 100 μm. **b.** Representative immunofluorescence images of two whole day 35 ENOs stained for ZO-1 with DAPI counterstained. Scale bars = 200 μm. **c.** Representative immunofluorescence image of day 50 ENO stained for DCX, NCAD, and SOX2 with DAPI counterstained. Scale bar = 100 μm. **d.** Representative immunofluorescence images of day 85 COs and ENOs stained for ZO-1, TUJ1 and SOX2 with DAPI counterstained. Scale bars = 100 μm. Images are representative of n=3 independent experiments.

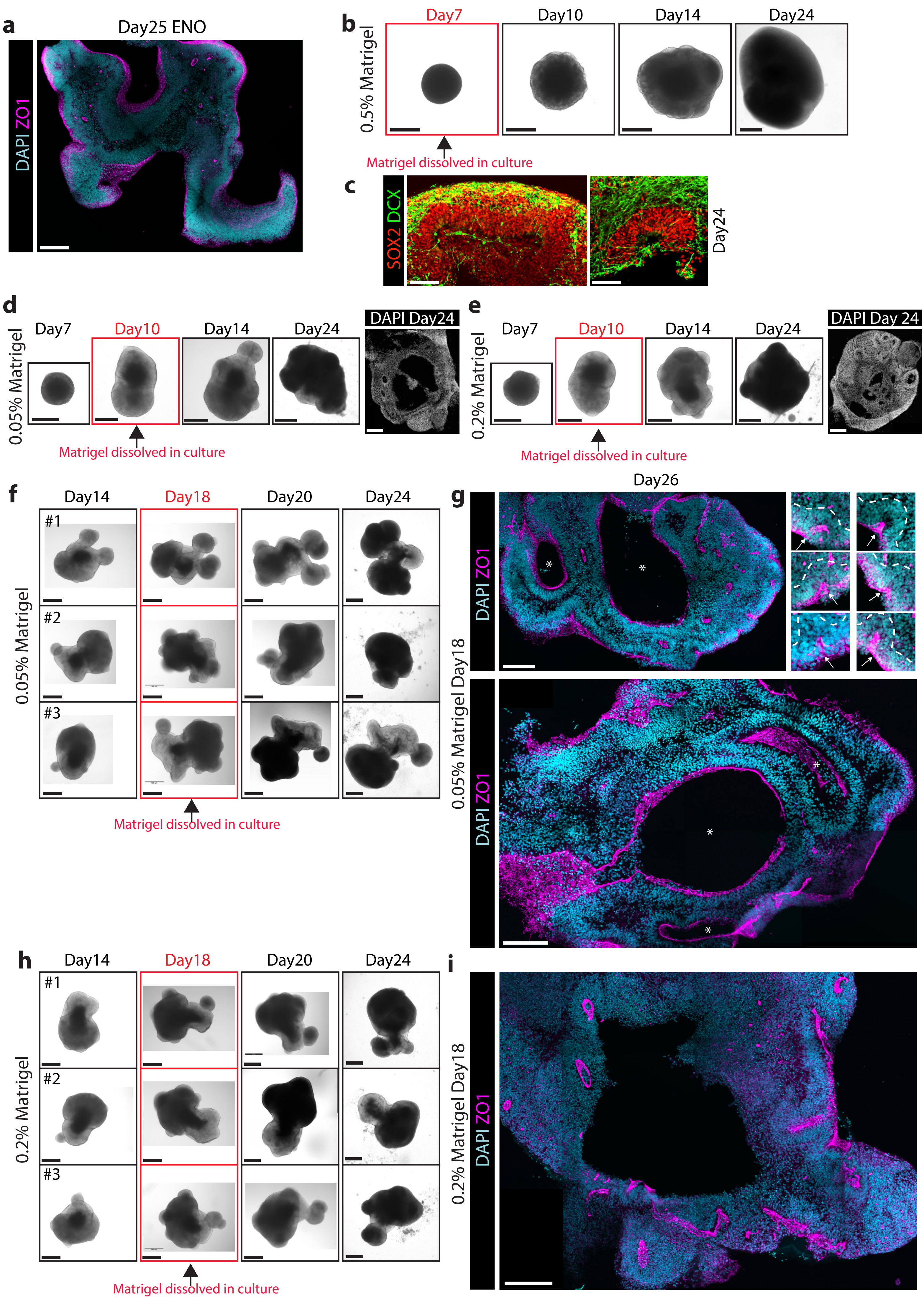

a
Day25 ENO
DAPI ZO1
b
0.5% Matrigel
Day7
Day10
Day14
Day24
Matrigel dissolved in culture
c
SOX2 DCX
Day24
d
0.05% Matrigel
Day7
Day10
Day14
Day24
DAPI Day24
Matrigel dissolved in culture
e
0.2% Matrigel
Day7
Day10
Day14
Day24
DAPI Day 24
Matrigel dissolved in culture
f
0.05% Matrigel
Day14
Day18
Day20
Day24
#1
#2
#3
Matrigel dissolved in culture
g
Day26
DAPI ZO1
0.05% Matrigel Day18
DAPI ZO1
h
0.2% Matrigel
Day14
Day18
Day20
Day24
#1
#2
#3
Matrigel dissolved in culture
i
0.2% Matrigel Day18
DAPI ZO1

Supplementary Figure 7. Effect of Matrigel on ENOs at earlier stages

a. Representative immunofluorescence image of a Day 24 ENO stained for ZO-1 with DAPI counterstained. Scale bar = 200 µm. **b.** Representative brightfield images of the modified ENOs protocol at the mentioned timepoints. Matrigel was dissolved in the medium (0.5%) starting from day 10. Scale bars = 500 µm. **c.** Representative immunofluorescence images of ENOs treated as described in b at day 24 stained for SOX2 and DCX. Scale bars = 50 µm. **d.** Representative brightfield images of the modified ENOs protocol at the mentioned timepoints and a DAPI staining on a sectioned organoid at day 24 on the right. Matrigel was dissolved in the medium (0.05%) starting from day 10. Scale bars = 500 µm. **e.** Representative brightfield images of the modified ENOs protocol at the mentioned timepoints and a DAPI staining on a sectioned organoid at day 24 on the right. Matrigel was dissolved in the medium (0.2%) starting from day 10. Scale bars = 500 µm. **f.** Representative brightfield images of the modified ENOs protocol at the mentioned timepoints. Matrigel was dissolved in the medium (0.05%) starting from day 18. Scale bars = 500 µm. **g.** Representative immunofluorescence images of two whole ENOs treated as described in f at day 26 stained for ZO-1 with DAPI counterstained. Asterisks (*) highlight enclosed ENO structures formed after Matrigel addition. Zoomed-in images highlight areas in which neuroepithelium structures pinch off into rosette-like structures. Scale bars = 200 µm. **h.** Representative brightfield images of the modified ENOs protocol at day 26 stained for ZO- 1 with DAPI counterstained. Matrigel was dissolved in the medium (0.2%) starting from day 18. Scale bar = 500 µm. **i.** Representative immunofluorescence images of a whole ENO treated as described in **h** at day 26 stained for ZO-1 with DAPI counterstained. Scale bar = 200 µm.

a

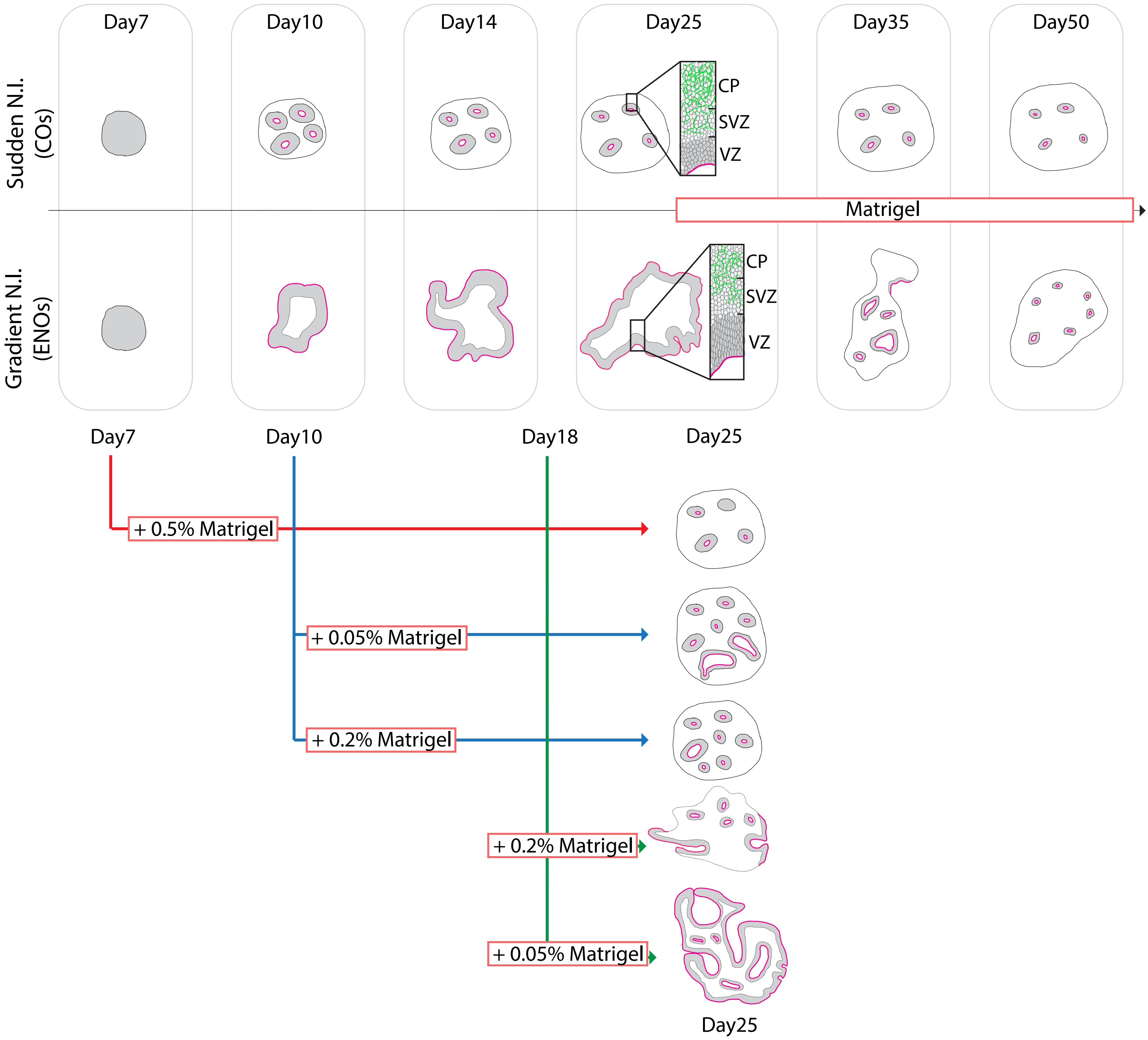
Day7
Day10
Day14
Day25
Day35
Day50
Sudden N.I. (COs)
Gradient N.I. (ENOs)
CP
SVZ
VZ
Matrigel
CP
SVZ
VZ
Day7
Day10
Day18
Day25
+ 0.5% Matrigel
+ 0.05% Matrigel
+ 0.2% Matrigel
+ 0.2% Matrigel
+ 0.05% Matrigel
Day25

Supplementary Figure 8. Schematic illustration of COs and ENOs morphology overtime

a. Schematic illustration of organoids generated with sudden N.I. (COs) and gradient N.I. (ENOs) at different timepoints. Organoids morphology is shown overtime for both protocols. The effect of adding Matrigel at different timepoints and concentrations is also represented, and the resulting morphology is graphically shown. Lines in magenta highlight the apical side of rosettes/neuroepithelium structures in COs and ENOs respectively.

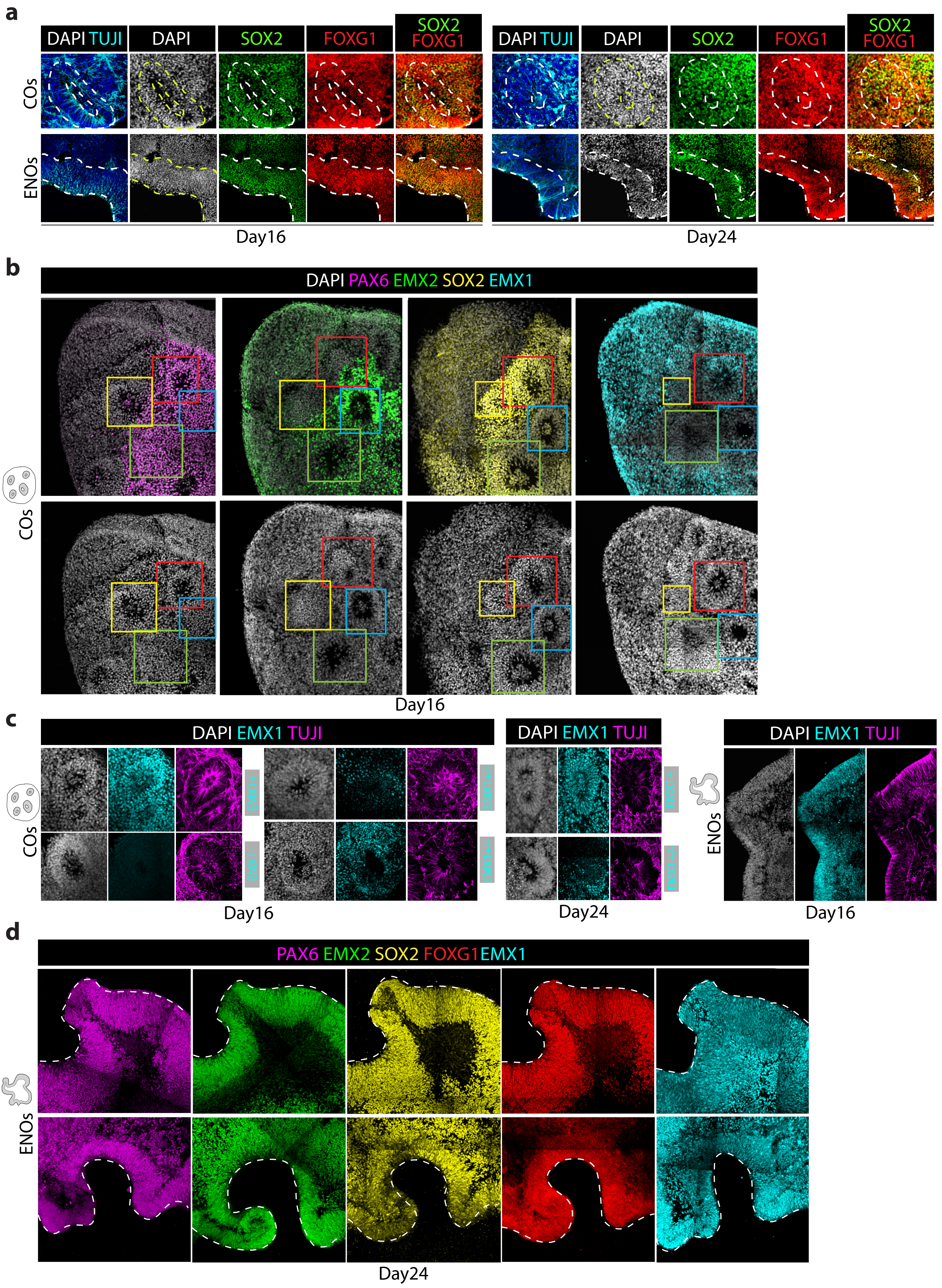

a
DAPI TUJI
DAPI
SOX2
FOXG1
SOX2 FOXG1
COs
ENOs
Day16
Day24
b
DAPI PAX6 EMX2 SOX2 EMX1
COs
Day16
c
DAPI EMX1 TUJI
EMX1+
EMX1-
EMX1+/-
COs
ENOs
Day16
Day24
d
PAX6 EMX2 SOX2 FOXG1 EMX1
ENOs
Day24

Supplementary Figure 9. Enhanced cortical identity in ENOs as compared to cortical organoids

a. Representative immunofluorescence images of COs and ENOs at day 16 and 24 stained for TUJ1, SOX2, and FOXG1 with DAPI counterstained. Dashed lines delineate the apical and basal perimeter of the rosettes and neuroepithelium structures in the different organoids. Scale bars = 100 μm. **b.** Representative immunofluorescence staining of consecutive sections of COs at day 16 stained for PAX6, EMX1, SOX2 and EMX2 with DAPI counterstained. Rosettes are highlighted in different colours to refer to the same rosette structure in the consecutive sections stained. Scale bars = 100 μm. **c.** Representative immunofluorescence images of COs (day 16 and 24) and ENOs (day 16) stained for EMX1 and TUJ1 with DAPI counterstained. Homogeneously EMX1 positive (EMX1+) and EMX1 negative (EMX1-) rosettes, as well as rosettes showing mixed EMX1 positive and negative portions (EMX1+/-) in COs are highlighted. **d.** Representative immunofluorescence images of consecutive sections of ENOs at day 24 stained for PAX6, EMX1, SOX2, EMX2, and FOXG1. Dashed lines delineate the basal perimeter of the neuroepithelium structures in ENOs. Scale bars = 100 μm.

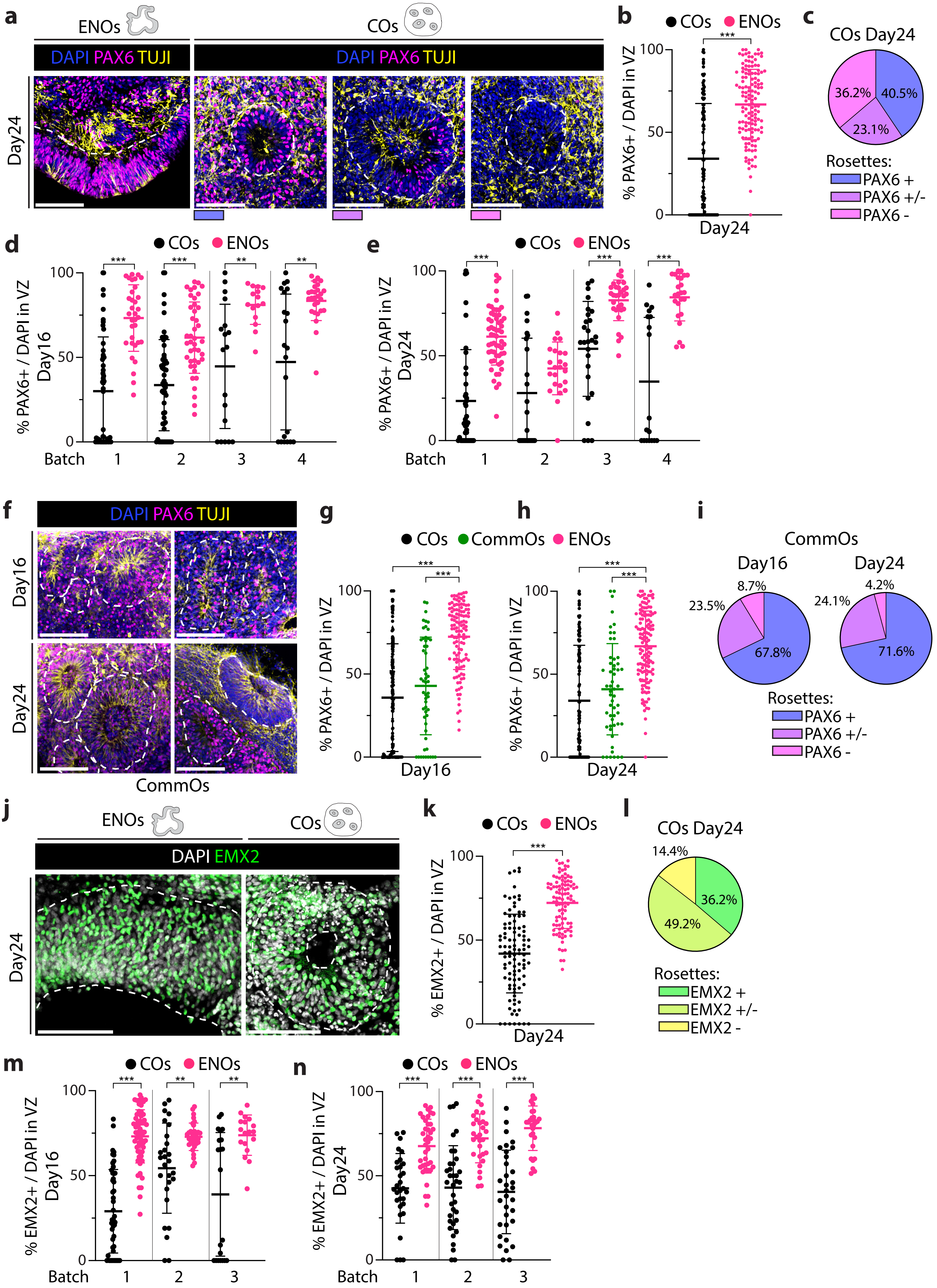

a
ENOs
COs
DAPI PAX6 TUJI
Day24
b
COs ENOs
% PAX6+ / DAPI in VZ
Day24
c
COs Day24
40.5%
23.1%
36.2%
Rosettes:
PAX6 +
PAX6 +/-
PAX6 -
d
% PAX6+ / DAPI in VZ Day16
Batch 1 2 3 4
e
% PAX6+ / DAPI in VZ Day24
f
Day16
Day24
CommOs
g
COs CommOs ENOs
Day16
h
Day24
i
CommOs
Day16
Day24
8.7%
23.5%
67.8%
4.2%
24.1%
71.6%
j
DAPI EMX2
k
% EMX2+ / DAPI in VZ
l
COs Day24
14.4%
36.2%
49.2%
EMX2 +
EMX2 +/-
EMX2 -
m
% EMX2+ / DAPI in VZ Day16
n
% EMX2+ / DAPI in VZ Day24

Supplementary Figure 10. Comparative forebrain regionality analysis of ENOs with cortical organoids protocols

a. Representative immunofluorescence images of ENOs and COs stained for PAX6 and TUJ1. Dashed lines delineate the basal perimeter of rosettes/neuroepithelium structures. Scale bars = 100 µm. **b.** Percentage of PAX6 positive cells over DAPI of COs and ENOs. Dots represent quantification in a different area of each measured rosette/neuroepithelium structure within the VZ. Mean ± SD is plotted. ***p < 0.001; Nonparametric Mann-Whitney U test. **c.** Pie chart of percentages of homogeneously PAX6 positive (PAX6+), negative (PAX6-) and mixed (PAX6+/-) rosettes in COs. **d-e.** Percentage of PAX6+ cells over DAPI of COs and ENOs, per batch at day 16 (**d**) and day 24 (**e**). Mean ± SD is plotted. **p < 0.01, ***p < 0.001; Nonparametric Mann-Whitney U test. **f.** Representative immunofluorescence images of CommOs stained for PAX6 and TUJ1. Dashed lines delineate the basal perimeter of rosettes/neuroepithelium structures. Scale bars = 100 µm. **g-h.** Percentage of PAX6 positive cells over DAPI of COs, CommOs and ENOs at day 16 (**g**) and day 24 (**h**). Mean ± SD is plotted. ***p < 0.001; Nonparametric Mann-Whitney U test. **i.** Pie chart of percentages of homogeneously PAX6 positive (PAX6+), negative (PAX6-) mixed (PAX6+/-) rosettes in CommOs at day 16 (left) and 24 (right). **j.** Representative immunofluorescence images ENOs and COs stained for EMX2. Dashed lines delineate apical and basal perimeter of neuroepithelium structures/rosettes. Scale bars = 100 µm. **k.** Percentage of EMX2+ cells over DAPI of COs and ENOs at day 24. Each dot represents quantification in a different area of each measured rosette/neuroepithelial structure within the VZ. Mean ± SD is plotted. A minimum of 3 different areas and 3 neuroepithelium structures/rosettes per organoid were quantified. ***p < 0.001; Nonparametric Mann-Whitney U test. **l.** Pie chart of percentages of homogeneously EMX2 positive (EMX2+) negative (EMX2-) mixed (EMX2+/-) rosettes in COs at day 24. **m-n.** Percentage of EMX2+ cells over DAPI of COs and ENOs per batch at day 16 (**m**) and day 24 (**n**). Mean ± SD is plotted. **p < 0.01, ***p < 0.001; Nonparametric Mann-Whitney U test. Images in **a, f** and **j** are representative of 3 independent experiments. Exact sample size and exact P values are provided in Source Data. Source data are provided as Source data file.

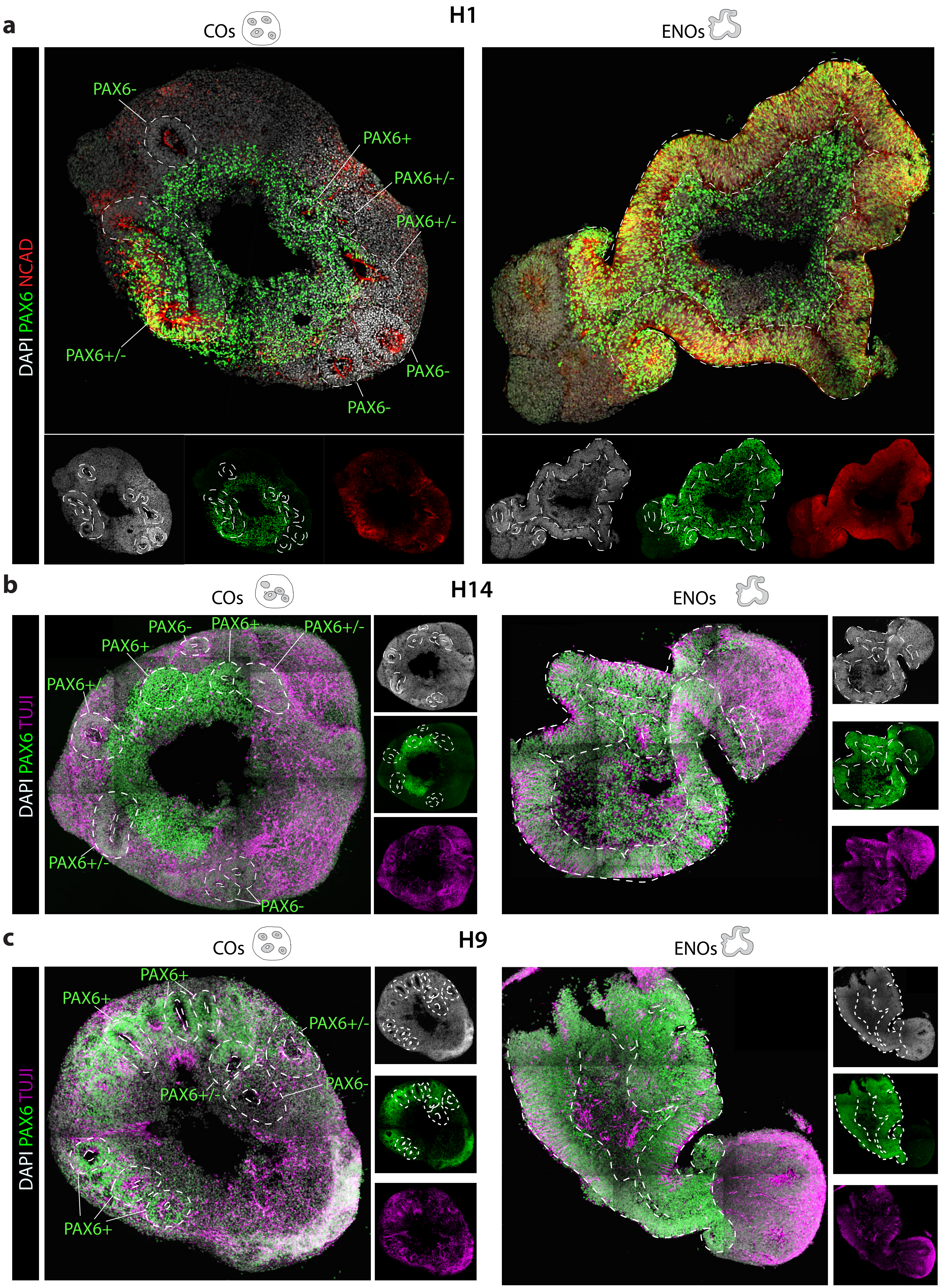

a
H1
COs
ENOs
DAPI PAX6 NCAD
PAX6-
PAX6+
PAX6+/-
PAX6+/-
PAX6+/-
PAX6-
PAX6-
b
H14
COs
ENOs
DAPI PAX6 TUJI
PAX6-
PAX6+
PAX6+/-
PAX6+
PAX6+/-
PAX6+/-
PAX6-
c
H9
COs
ENOs
DAPI PAX6 TUJI
PAX6+
PAX6+
PAX6+/-
PAX6-
PAX6+/-
PAX6+

Supplementary Figure 11. Enhanced cortical identity in ENOs generated from different hESC lines

a. Representative immunofluorescence images of a whole CO and ENO formed with H1 hESC line at day 16 stained for PAX6 and NCAD with DAPI counterstained. Dashed lines delineate the apical and basal perimeter of rosettes and neuroepithelium structures of the different organoids. Homogeneously PAX6 positive (PAX6+) and PAX6 negative (PAX6-) rosettes, as well as rosettes showing mixed PAX6 positive and negative portions (PAX6+/-) in COs are highlighted. Scale bars = 500 μm.

b. Representative immunofluorescence images of a whole CO and ENO formed with H14 hESC line at day 16 stained for PAX6 and TUJ1 with DAPI counterstained. Dashed lines delineate the apical and basal perimeter of rosettes and neuroepithelium structures of the different organoids. Homogeneously PAX6 positive (PAX6+) and PAX6 negative (PAX6-) rosettes, as well as rosettes showing mixed PAX6 positive and negative portions (PAX6+/-) in COs are highlighted. Scale bars = 500 μm.

c. Representative immunofluorescence images of a whole CO and ENO formed with the H9 hESC line at day 16 stained for PAX6 and TUJ1 with DAPI counterstained. Dashed lines delineate the apical and basal perimeter of rosettes and neuroepithelium structures of the different organoids. Homogeneously PAX6 positive (PAX6+) and PAX6 negative (PAX6-) rosettes, as well as rosettes showing mixed PAX6 positive and negative portions (PAX6+/-) in COs are highlighted. Scale bars = 500 μm.

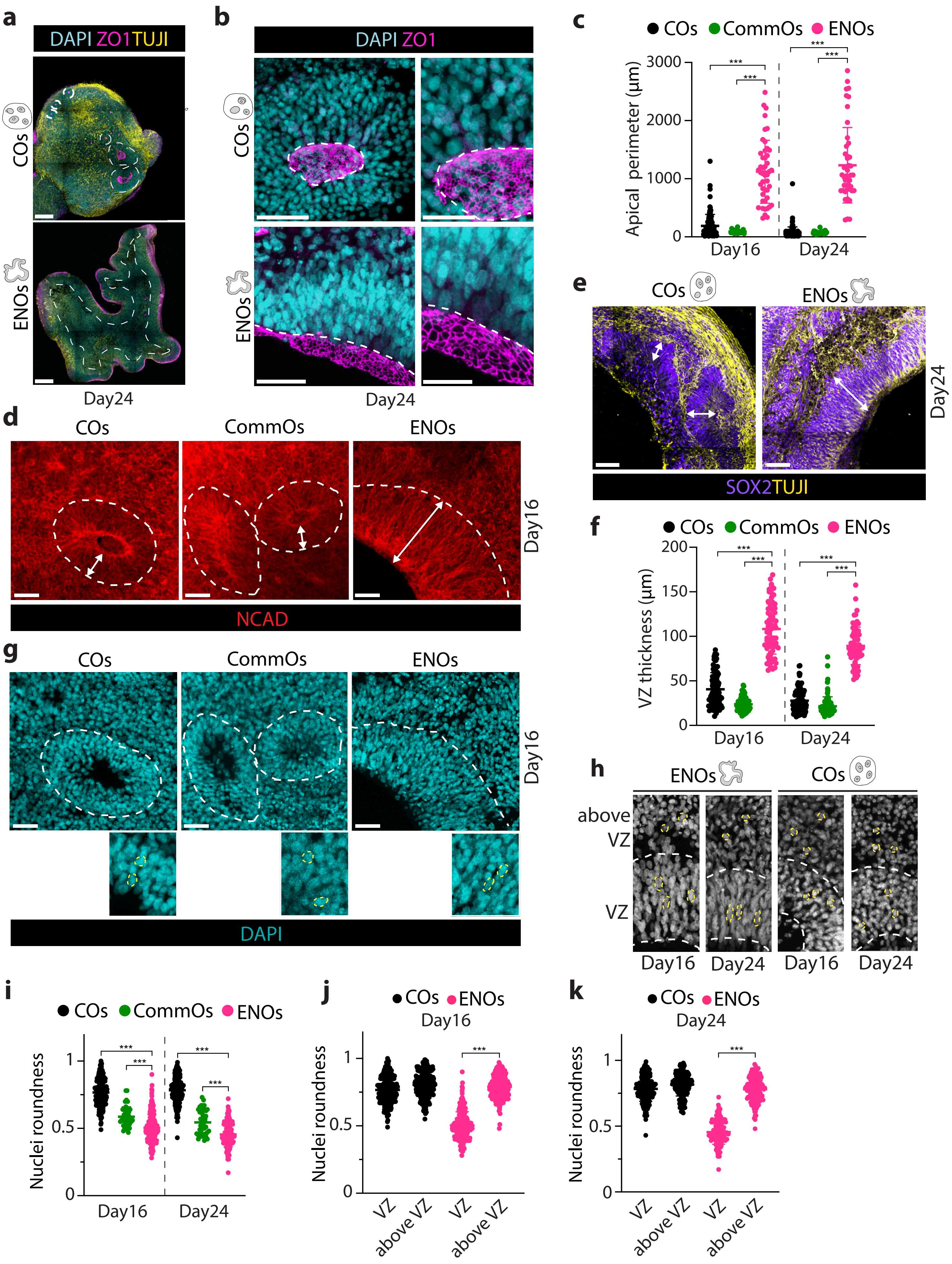

a
DAPI ZO1TUJI
COs
ENOs
Day24
b
DAPI ZO1
COs
ENOs
Day24
c
COs CommOs ENOs
Apical perimeter (µm)
3000
2000
1000
0
***
***
***
***
Day16
Day24
d
COs
CommOs
ENOs
Day16
NCAD
e
COs
ENOs
Day24
SOX2TUJI
f
COs CommOs ENOs
VZ thickness (µm)
200
150
100
50
0
***
***
***
***
Day16
Day24
g
COs
CommOs
ENOs
Day16
DAPI
h
ENOs
COs
above VZ
VZ
Day16
Day24
Day16
Day24
i
COs CommOs ENOs
Nuclei roundness
1
0.5
0
***
***
***
***
Day16
Day24
j
COs ENOs
Day16
Nuclei roundness
1
0.5
0
***
VZ
above VZ
VZ
above VZ
k
COs ENOs
Day24
Nuclei roundness
1
0.5
0
***
VZ
above VZ
VZ
above VZ

Supplementary Figure 12. Comparative morphological analysis of ENOs with cortical organoid protocols

a. Representative immunofluorescence images of COs and ENOs stained for ZO-1 and TUJ1 with DAPI counterstained. Scale bars = 250 µm. **b.** Representative immunofluorescence images of COs and ENOs stained for ZO-1 with DAPI counterstained. Scale bars = 50 µm for low mag and 25 µm for high mag. **c.** Quantification of the apical perimeter of rosettes/neuroepithelium structures of COs, CommOs, and ENOs. Dots represent individual rosettes/neuroepithelium structures. Mean ± SD is plotted. ***p < 0.001; Unpaired t-test. **d.** Representative immunofluorescence images of COs, CommOs and ENOs stained for NCAD. Arrows point at the VZ. Scale bars = 50 µm. **e.** Representative immunofluorescence images of COs and ENOs stained for SOX2 and TUJ1 with DAPI counterstained. Arrows point at the VZ. Scale bars = 50 µm. **f.** Quantification of the VZ thickness of the rosettes/neuroepithelium structures of COs, CommOs, and ENOs at day 16 and 24, based on distance between apical and basal side. Multiple rosettes/neuroepithelium structures are measured per organoid. Each dot represents average of three measurements for each individual neuroepithelium/rosette structure and mean ± SD is plotted. ***p < 0.001; Unpaired t- test. **g.** Representative DAPI stainings of COs, CommOs, and ENOs at day 16. Dashed lines in white delineate the basal perimeter of rosettes and neuroepithelium structures in the different organoids. Dashed lines in yellow delineate nuclei shape. Scale bars = 50 µm. **h.** Representative DAPI stainings of rosettes/neuroepithelium structures of COs and ENOs. Dashed lines in white delineate the VZ and above VZ areas. Dashed lines in yellow delineate nuclei shape. **i.** Quantification of the nuclei roundness measured for nuclei found in the VZ of rosettes/neuroepithelial structures of COs and ENOs. Dots represent individual nuclei. Mean ± SD is plotted. ***p < 0.001; Unpaired t-test. **j.** Quantification of nuclei roundness measured for nuclei found in the VZ and above the VZ of COs and ENOs. Dots represent individual nuclei. Mean ± SD is plotted. ***p < 0.001; Unpaired t-test. **k.** Quantification of nuclei roundness measured for cells in VZ and above of COs and ENOs. Dots represent individual nuclei. Mean ± SD is plotted. ***p < 0.001; Unpaired t-test. Dashed lines delineate the basal **(a)** and apical **(b)** perimeter of rosettes and neuroepithelium structures in the different organoids. Exact sample size for **c, f, f, i, j** and **k** and exact P values are provided in Source Data. Source data are provided as Source data file.

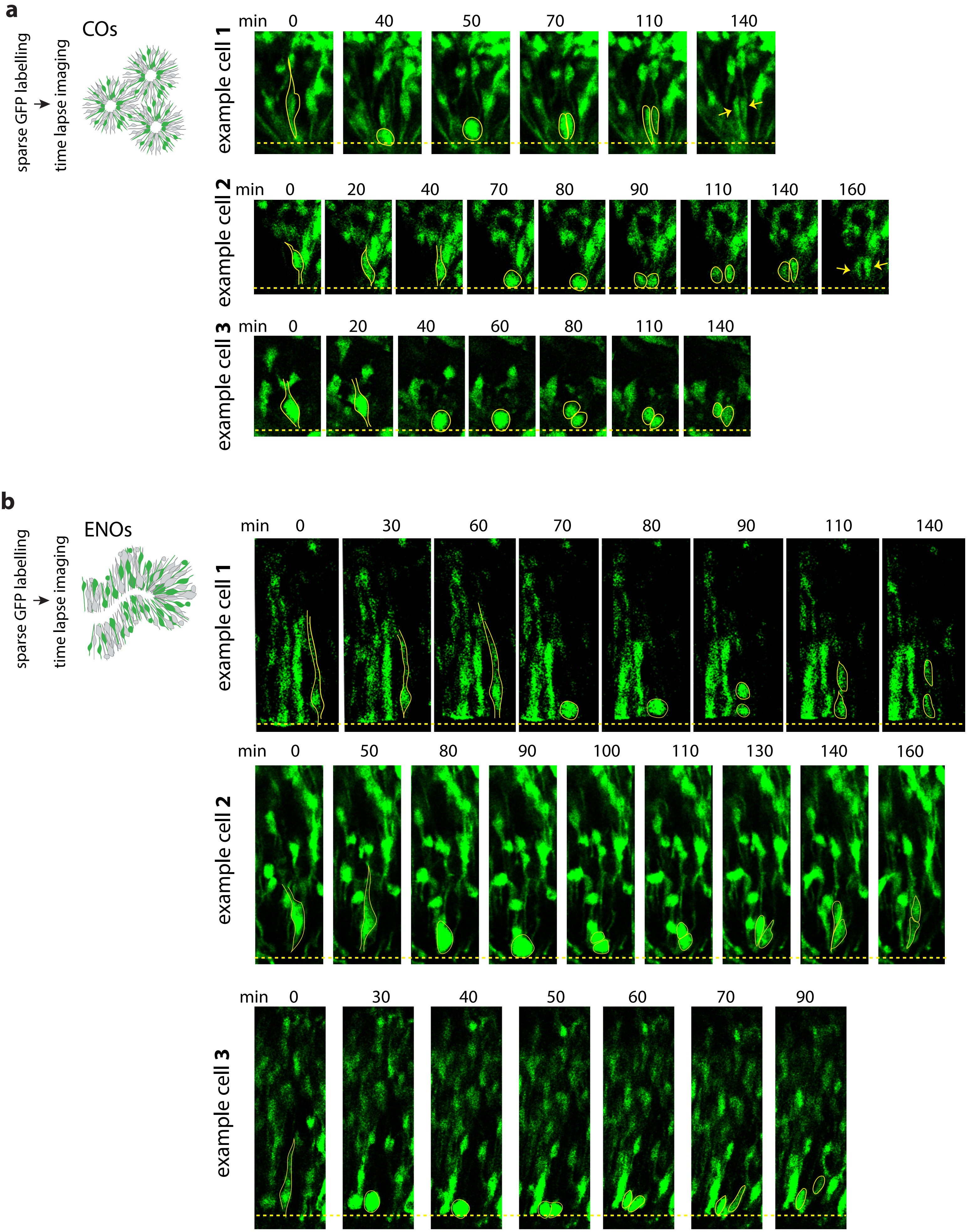
a
sparse GFP labelling
time lapse imaging
COs
example cell 1
min 0 40 50 70 110 140
example cell 2
min 0 20 40 70 80 90 110 140 160
example cell 3
min 0 20 40 60 80 110 140
b
sparse GFP labelling
time lapse imaging
ENOs
example cell 1
min 0 30 60 70 80 90 110 140
example cell 2
min 0 50 80 90 100 110 130 140 160
example cell 3
min 0 30 40 50 60 70 90

Supplementary Figure 13. Distinct mode of interkinetic nuclear migration in ENOs as compared to COs

a. Representative still frames of live imaging of GFP-labelled cells undergoing mitosis in COs at day 14. Dashed lines represent apical side. n = 3 cell divisions are shown.

b. Representative still frames of live imaging of GFP-labelled cells undergoing mitosis in ENOs at day 14. Dashed lines represent apical side. n = 3 cell divisions are shown.

Supplementary Table 1. Primary and secondary antibodies used in this study

Primary antibodies

Antibody	Dilution	Source	Cat. Number
Anti-PAX6	1:1000	Rabbit Polyclonal	Covance catalog, #PRB-278P
Anti-SOX2	1:1000	Rabbit Polyclonal	Sigma, #AB5603
Anti-Ki67	1:1000	Rat	Fisher, #14-5698-82
Anti-Doublecortin	1:1000	Guinea pig	Sigma, #AB2253
Anti-N-cadherin	1:300	Mouse monoclonal	Santa Cruz, #SC59987
Anti-ZO-1	1:500	Rabbit	Invitrogen, #402200
Anti-CTIP2	1:200	Rat	Abcam, #ab18465
Anti-BRN2	1:300	Mouse	Santa Cruz, #SC-393324AC
Anti-SATB2	1:50	Mouse	Abcam, #ab51502
Anti-Tbr2	1:200	Rabbit	Abcam, #ab23345
Anti-Auts2	1:200	Mouse	Thermo Fisher, #MA5-31447
Anti-Sox5	1:200	Rabbit	Abcam, #ab94396
Anti-Emx2	1:200	Rabbit	Novus Bio, #NBP2-39052
Anti-SOX2	1:1000	Rat	eBioScience, #14-9811-82
Anti-S100B	1:500	Rabbit	Sigma Aldrich, #HPA008393
Anti-TBR2	1:500	Mouse	R&D systems (BioTechne) # MAB6166-SP
Anti-EMX1	1:100	Rabbit	Sigma Aldrich, #HPA006421
Anti-FOXG1	1:1000	Rabbit	Abcam, #ab18259

Secondary antibodies

Antibody	Dilution	Cat. number
Alexa Fluor 488 donkey anti-rabbit IgG (H+L)	1:1000	Life technologies, #A21206
Alexa Fluor 488 goat anti-guinea pig IgG (H+L)	1:1000	Invitrogen, #A11073

Alexa Fluor 568 donkey anti-mouse IgG (H+L)	1:1000	Life technologies, #A10037
Alexa Fluor 647 donkey anti-rabbit IgG (H+L)	1:1000	Life technologies, #A31573
Alexa Fluor 647 goat anti-rat IgG (H+L)	1:1000	Life technologies, #A21247

Supplementary Table 2. qPCR primers used in this study

**Target**	**Sequence**
ACTB Fw	GAAAATCTGGCACCACACCT
ACTB Rv	TAGCACAGCCTGGATAGCAA
TBR1 Fw	GGGCTCACTGGATGCGCCAAG
TBR1 Rv	TCCGTGCCGTCCTCGTTCACT
TBR2 Fw	CGGCCTCTGTGGCTCAAA
TBR2 Rv	AAGGAAACATGCGCCTGC
EMX1 Fw	GAGACGCAGGTGAAGGTGT
EMX1 Rv	GTTGATGTGATGGGAGCCCT
EMX2 Fw	CTGGAACACGCCTTTGAGA
EMX2 Rv	CCAGCTTCTGCCTTTTGAAC
PAX6 Fw	TTGCCCGAGAAAGACTAGCA
PAX6 Rv	TCTCCATTTGGCCCTTCGATTA
FOXG1 Fw	CCTGCCCTGTGAGTCTTTAAG
FOXG1 Rv	GTTCACTTACAGTCTGGTCCC
SOX2 Fw	GAGCTTTGCAGGAAGTTTGC
SOX2 Rv	GCAAGAAGCCTCTCCTTGAA
CENPF Fw	CTCTCCCGTCAACAGCGTTC
CENPF Rv	CTCTCCCGTCAACAGCGTTC
NCAD Fw	CCTCCAGAGTTTACTGCCATGAC
NCAD Rv	GTAGGATCTCCGCCACTGATTC
BRACH Fw	CCTTCAGCAAAGTCAAGCTCACC
BRACH Rv	TGAACTGGGTCTCAGGGAAGCA
NESTIN Fw	TCAAGATGTCCCTCAGCCTGGA
NESTIN Rv	AAGCTGAGGGAAGTCTTGGAGC
ECAD Fw	GCCTCCTGAAAAGAGAGTGGAAG
ECAD Rv	TGGCAGTGTCTCTCCAAATCCG
SOX17 Fw	ACGCTTTCATGGTGTGGGCTAAG
SOX17 Rv	GTCAGCGCCTTCCACGACTTG

Supplementary Table 3. Overview of the number of organoids and batches used throughout the study

	DAY7	*DAY10*	*DAY14*	*DAY20*	*DAY25*
**ENO H1**	73	72	72	41	36
**CO H1**	46	46	49	40	35
**ENO H9**	10	21	21	13	12
**CO H9**	22	32	33	23	23
**ENO H14**	31	31	31	17	16
**CO H14**	13	13	13	8	7

The number of organoids analysed in **Figure 1d-e**. A total number of n = 6, 3 and 2 batches for both ENOs and COs were analysed for the H1, H9, and H14 hESC lines, respectively.

	DAY7	*DAY10*	*DAY14*	*DAY20*	*DAY25*
CO H1	31	34	37	29	27
FULL SB43 H1	36	36	46	35	25
ENO H1	72	74	73	44	31
NO SB43 H1	23	33	42	33	29

The number of organoids analysed in **Figure 2d-e**. A total number of n = 4 batches were analysed.

	DAY7	*DAY10*	*DAY14*	*DAY20*	*DAY25*
ENO H1	73	72	72	41	36
CO H1	46	46	49	40	35
COMMO H1	12	6	6	7	7

The number of organoids analysed in **Supplementary Figure 1d-e** and **1g**. A total number of n = 6 batches for ENOs and COs and n = 2 batches for CommOs were analyzed.

	DAY7	*DAY10*	*DAY14*	*DAY20*	*DAY25*
CO H9	55	65	66	44	41
EN0 H9	10	21	21	13	12
NO SB43 H9	44	48	49	29	29

The number of organoids analysed in **Supplementary Figure 4c-d**. A total number of n = 3 batches for ENOs and n = 4 batches for COs and no SB43 were analysed using the H9 hESC line.

	DAY7	*DAY10*	*DAY14*	*DAY20*	*DAY25*
CO H14	13	13	13	8	7
EN0 H14	31	31	31	17	16
NO SB43 H14	31	31	31	17	17

The number of organoids analysed in **Supplementary Figure 4e-f**. A total number of n = 2 batches were analysed using the H14 hESC line.
