# Supplementary material for: Temporal morphogen gradient-driven neural induction shapes single expanded neuroepithelium brain organoids with enhanced cortical identity
Source: Nat Commun. 2023 Nov 28;14:7361. doi: 10.1038/s41467-023-43141-1 (PMC10684874; doi:10.1038/s41467-023-43141-1)
Supplement: Supplementary file 3 — Description of Additional Supplementary Files [file 41467_2023_43141_MOESM3_ESM.pdf]

Description of Additional Supplementary Files

Supplementary Movie 1: 3D rendering of a whole ENO at day 24 stained for PAX6, TUJ1, and ZO-1.

Supplementary Movie 2: 3D rendering of a whole ENO at day 24 stained for N-CAD and PAX6.
