# Supplementary material for: Temporal morphogen gradient-driven neural induction shapes single expanded neuroepithelium brain organoids with enhanced cortical identity
Source: Nat Commun. 2023 Nov 28;14:7361. doi: 10.1038/s41467-023-43141-1 (PMC10684874; doi:10.1038/s41467-023-43141-1)
Supplement: Supplementary file 6 — Reporting Summary [file 41467_2023_43141_MOESM6_ESM.pdf]

nature portfolio

Corresponding author(s): Benedetta Artegiani, Delilah Hendriks

Last updated by author(s): Oct 24, 2023

Reporting Summary

Nature Portfolio wishes to improve the reproducibility of the work that we publish. This form provides structure for consistency and transparency in reporting. For further information on Nature Portfolio policies, see our Editorial Policies and the Editorial Policy Checklist.

Statistics

For all statistical analyses, confirm that the following items are present in the figure legend, table legend, main text, or Methods section.

n/a	Confirmed	
☐	☒	The exact sample size (*n*) for each experimental group/condition, given as a discrete number and unit of measurement
☐	☒	A statement on whether measurements were taken from distinct samples or whether the same sample was measured repeatedly
☐	☒	The statistical test(s) used AND whether they are one- or two-sided<br>*Only common tests should be described solely by name; describe more complex techniques in the Methods section.*
☒	☐	A description of all covariates tested
☒	☐	A description of any assumptions or corrections, such as tests of normality and adjustment for multiple comparisons
☐	☒	A full description of the statistical parameters including central tendency (e.g. means) or other basic estimates (e.g. regression coefficient) AND variation (e.g. standard deviation) or associated estimates of uncertainty (e.g. confidence intervals)
☐	☒	For null hypothesis testing, the test statistic (e.g. *F*, *t*, *r*) with confidence intervals, effect sizes, degrees of freedom and *P* value noted<br>*Give P values as exact values whenever suitable.*
☒	☐	For Bayesian analysis, information on the choice of priors and Markov chain Monte Carlo settings
☒	☐	For hierarchical and complex designs, identification of the appropriate level for tests and full reporting of outcomes
☒	☐	Estimates of effect sizes (e.g. Cohen's *d*, Pearson's *r*), indicating how they were calculated

Our web collection on statistics for biologists contains articles on many of the points above.

Software and code

Policy information about availability of computer code

Data collection	Leica LAS X (v3.5.7).
Data analysis	Fiji (v2.0.0), Leica LAS X (v3.5.7), Microsoft Excel 2020, GraphPad Prism (v8.2.0), Illustrator, Photoshop CS4.

For manuscripts utilizing custom algorithms or software that are central to the research but not yet described in published literature, software must be made available to editors and reviewers. We strongly encourage code deposition in a community repository (e.g. GitHub). See the Nature Portfolio guidelines for submitting code & software for further information.

Data

Policy information about availability of data

All manuscripts must include a data availability statement. This statement should provide the following information, where applicable:

- Accession codes, unique identifiers, or web links for publicly available datasets
- A description of any restrictions on data availability
- For clinical datasets or third party data, please ensure that the statement adheres to our policy

A data availability statement is present in the manuscript. All data associated with this manuscript can be provided upon reasonable request.

Human research participants

Policy information about studies involving human research participants and Sex and Gender in Research.

Reporting on sex and gender	No human research participants were involved in this study.
Population characteristics	N/A.
Recruitment	N/A.
Ethics oversight	N/A.

Note that full information on the approval of the study protocol must also be provided in the manuscript.

Field-specific reporting

Please select the one below that is the best fit for your research. If you are not sure, read the appropriate sections before making your selection.

[x] Life sciences [ ] Behavioural & social sciences [ ] Ecological, evolutionary & environmental sciences

For a reference copy of the document with all sections, see nature.com/documents/nr-reporting-summary-flat.pdf

Life sciences study design

All studies must disclose on these points even when the disclosure is negative.

Sample size	No sample size calculation was performed to predetermine sample sizes. The sample sizes were in the same range of other studies in the field. Results were validated across multiple independent organoid batches and using multiple organoids. The n is indicated in the figure legends.
Data exclusions	No data were excluded.
Replication	Experiments were confirmed across multiple independent experiments, across multiple organoids batches and with multiple organoids each.
Randomization	Not applicable. There are no experiments that require randomization and therefore no randomization was performed.
Blinding	The researchers were not blinded since no experiments needed blinding for analysis.

Reporting for specific materials, systems and methods

We require information from authors about some types of materials, experimental systems and methods used in many studies. Here, indicate whether each material, system or method listed is relevant to your study. If you are not sure if a list item applies to your research, read the appropriate section before selecting a response.

Materials & experimental systems

n/a	Involved in the study	
[ ]	[x]	Antibodies
[ ]	[x]	Eukaryotic cell lines
[x]	[ ]	Palaeontology and archaeology
[x]	[ ]	Animals and other organisms
[x]	[ ]	Clinical data
[x]	[ ]	Dual use research of concern

Methods

n/a	Involved in the study	
[x]	[ ]	ChIP-seq
[x]	[ ]	Flow cytometry
[x]	[ ]	MRI-based neuroimaging

Antibodies

Antibodies used	Primary: rabbit anti-Pax-6 (previously Covance catalog #PRB-278P), Biolegend, dilution 1:1000; rabbit anti-Sox2, AB5603, Sigma-Aldrich, dilution 1:1000; rat anti-Ki67 (SolA15), 14-5698-82, Thermo Fisher, dilution 1:1000; guinea pig anti-Doublecortin, AB2253, Sigma-Aldrich, dilution 1:1000; mouse anti-N-cadherin (13A9), sc-59987, Santa Cruz, dilution 1:300; rabbit anti-ZO1, 40-2200, Thermo Fisher, dilution 1:500; rabbit anti-TBR1, ab31940, Abcam, dilution 1:500; rat anti-Ctip2 (25B6), ab18465, Abcam, dilution 1:200; mouse anti-Brn-2/BRN2/POU3F2 (B-2), sc-393324, Santa Cruz, dilution 1:300; mouse anti-SATB2 (SATBA4B10), ab51502, Abcam, dilution 1:50; rabbit anti-TBR2/Eomes, ab23345, Abcam, dilution 1:200; rabbit anti-PTPRZ, PA5-101832, Thermo Fisher, dilution 1:200; mouse anti-Hop (E-1), sc-398703, Santa Cruz, dilution 1:200; mouse anti-AUTS2 (CL7084), MA5-31447, Thermo Fisher, dilution 1:200; rabbit anti-SOX5, ab94396, Abcam, dilution 1:200; rabbit anti-EMX2, NBP2-39052, Novus Biologicals, dilution 1:200.

	Secondary: Alexa Fluor 488 donkey anti-rabbit, A21206; Alexa Fluor 488 goat anti-guinea pig, A11073; Alexa Fluor 568 donkey anti-mouse, A10037; Alexa Fluor 647 donkey anti-rabbit, A31573; Alexa Fluor 647 goat anti-rat, A21247. All secondary antibodies were purchased from Thermo Fisher, dilution 1:1000.
Validation	All antibodies were validated by the manufacturers and have been used across multiple publications.<br>Rabbit anti-Pax-6 (previously Covance catalog #PRB-278P), Biolegend (https://www.biolegend.com/nl-be/products/purified-anti-pax-6-antibody-11511). Citations: e.g. PMID: 28445462; PMID: 22492355.<br>Rabbit anti-Sox2, AB5603, Sigma-Aldrich (https://www.sigmaaldrich.com/NL/en/product/mm/ab5603). Anti-SOX2 Antibody, Cat. No. AB5603, is a highly specific rabbit polyclonal antibody SOX2 and has been tested for use in Immunocytochemistry, and Immunohistochemistry (Paraffin), and Western Blotting. Citations: e.g. PMID: 22407749; PMID: 22675207.<br>Rat anti-Ki67 (SolA15), 14-5698-82, Thermo Fisher (https://www.thermofisher.com/antibody/product/Ki-67-Antibody-clone-SolA15-Monoclonal/14-5698-82). This Antibody was verified by Cell treatment to ensure that the antibody binds to the antigen stated. Citations: e.g. PMID: 34525348, PMID: 34100459.<br>Guinea pig anti-Doublecortin, AB2253, Sigma-Aldrich (https://www.sigmaaldrich.com/NL/en/product/mm/ab2253?gclid=EAIaIQobChMI0LeR1OXO_AIVjOd3Ch0g0gSfEAAYASAAEgJYkPD_BwE&gclsrc=aw.ds). Citations: e.g. PMID: 26387477; PMID: 28743634.<br>Mouse anti-N-cadherin (13A9), sc-59987, Santa Cruz (https://www.scbt.com/p/n-cadherin-antibody-13a9?requestFrom=search). Citations: e.g. PMID: 35093077; PMID: 36499014.<br>Rabbit anti-ZO1, 40-2200, Thermo Fisher (https://www.thermofisher.com/antibody/product/ZO-1-Antibody-Polyclonal/40-2200). This Antibody was verified by Cell treatment to ensure that the antibody binds to the antigen stated. Citations: e.g. PMID: 31130514; PMID: 31601814.<br>Rabbit anti-TBR1, ab31940, Abcam (https://www.abcam.com/tbr1-antibody-ab31940.html). Citations: e.g. PMID: 33501759; PMID: 33470930.<br>Rat anti-Ctip2 (25B6), ab18465, Abcam (https://www.abcam.com/ctip2-antibody-25b6-ab18465.html). Citations: e.g. PMID: 33723434; PMID: 33765444.<br>Mouse anti-Brn-2/BRN2/POU3F2 (B-2), sc-393324, Santa Cruz (https://www.scbt.com/p/brn-2-antibody-b-2?requestFrom=search). Citations: e.g. PMID: 35172154; PMID: 35254502.<br>Mouse anti-SATB2 (SATBA4B10), ab51502, Abcam (https://www.abcam.com/satb1--satb2-antibody-satba4b10-c-terminal-ab51502.html). Positive control used for ICC are HT10180 cells. Citations: e.g. PMID: 33431859; PMID: 33723434.<br>Rabbit anti-TBR2/Eomes, ab23345, Abcam (https://www.abcam.com/tbr2--eomes-antibody-ab23345.html). Citations: e.g. PMID: 33378662; PMID: 33407494.<br>Rabbit anti-PTPRZ, PA5-101832, Thermo Fisher (https://www.thermofisher.com/antibody/product/PTPRZ-Antibody-Polyclonal/PA5-101832). Citations: e.g. PMID: 33815059.<br>Mouse anti-Hop (E-1), sc-398703, Santa Cruz (https://www.scbt.com/p/hop-antibody-e-1). Citations: e.g. PMID: 35714603; PMID: 35732132.<br>Mouse anti-AUTS2 (CL7084), MA5-31447, Thermo Fisher (https://www.thermofisher.com/antibody/product/AUTS2-Antibody-clone-CL7084-Monoclonal/MA5-31447).<br>Rabbit anti-SOX5, ab94396, Abcam (https://www.abcam.com/sox5-antibody-ab94396.html). Citations: e.g. PMID: 33473108; PMID: 30503141.<br>Rabbit anti-EMX2, NBP2-39052, Novus Biologicals (https://www.novusbio.com/products/emx2-antibody_nbp2-39052). Validated by orthogonal strategies.

Eukaryotic cell lines

Policy information about cell lines and Sex and Gender in Research

Cell line source(s)	H1 Human ES cell line (WA01), Wicell
Authentication	Not applicable. Authentication performed by vendor.
Mycoplasma contamination	ES cells were monthly tested for mycoplasma contamination and tested negative without exception.
Commonly misidentified lines<br>(See ICLAC register)	N/A.
